# Perceptions of Heat Risk to Health: A Qualitative Study of Professional Bus Drivers and Their Managers in Jinan, China

**DOI:** 10.3390/ijerph110201520

**Published:** 2014-01-28

**Authors:** Lin Zhou, Zheng Xin, Li Bai, Fangjun Wan, Yongming Wang, Shaowei Sang, Shouqin Liu, Ji Zhang, Qiyong Liu

**Affiliations:** 1 State Key Laboratory for Infectious Diseases Prevention and Control, National Institute for Communicable Disease Control and Prevention, Chinese Center for Disease Control and Prevention, 155 Changbai Road, Changping District, Beijing 102206, China; E-Mails: zhoulin.scu@163.com (L.Z.); yan_lili407@126.com (L.B.); wanfangjun1989@163.com (F.W.); sangshaowei1@163.com (S.S.); 2 School of Public Health, Chinese Academy of Medical Sciences & Peking Union Medical College (CAMS & PUMC), Beijing 100073, China; 3 Jinan Municipal Center for Disease Control and Prevention, 2 Weiliu Road, Jinan 250021, Shandong, China; E-Mails: xinzheng121@163.com (Z.X.); wangyongming1211@tom.com (Y.W.); liushouqin@sina.com (S.L.); zhangji1967@hotmail.com (J.Z.); 4 Shandong University Climate Change and Health Center, 44 Wenhua Road, Jinan 250012, Shandong, China

**Keywords:** occupational driver, heat, risk perception, public health

## Abstract

Summer extreme heat threatens the health of individuals, especially persons who are involved in outdoor activities. Ensuring the normal function of a city, bus drivers are among those who participate in outdoor physical activities and are exposed to excessive heat in hot summer weather. This qualitative study was performed to explore professional bus drivers’ in-depth views of extreme heat risks to their health, and ultimately develop targeted advice and policy interventions for city bus drivers. An interview-based study was performed among professional bus drivers in Jinan, China, including four focus groups with professional bus drivers (*n* = 37) and three interviews with their managers (*n* = 14). Five central themes or categories from the bus driver interviews were found: concerns about summer heat; health effects related to extreme heat; adaptive measures; barriers in implementing these adaptive measures; and suggested interventions. The beneficial role of cooling facilities (particularly air-conditioning) during extreme heat are addressed. The barriers not only impede the implementation of behavioral adaptive measures but also enhance the negative attitudes of bus drivers towards their effectiveness. The responsibilities of managers in promoting preventive actions are addressed.

## 1. Introduction

Summer extreme heat threatens the health of individuals, especially persons with pre-existing illnesses, and those who are involved in outdoor activities [1,2]. Dehydration, heat exhaustion, heat cramps and heat stroke are commonly observed heat-related conditions during extreme heat episodes [3]. In addition to adverse health impacts, excessive heat can claims numerous people’s lives. During a severe heat wave in New South Wales, Australia in 2011, all-cause mortality and ambulance calls increased by 13% and 14%, respectively [4]. A heat wave that occurred in Europe in the summer of 2003 caused a large number of heat-related deaths, including nearly 15,000 in France alone [5]. Work requiring exposure to extremely hot weather (e.g., construction, road paving, power line maintenance and other outdoor municipal work) can be very dangerous [6]. Compared with people with other forms of employment, outdoors workers have a 20-fold increased rate of heat-related death [7]. To ensure the normal function of a city, bus drivers are among those who participate in outdoor physical activities and are exposed to excessive heat in hot summer weather [6,8].

Urban bus drivers are more likely to be affected by uncomfortable heat, as they spend most of time sitting in the narrow cab and driving on route. They work under great psychological pressure [9]. Bus driving is mentally demanding because it involves keeping to a tight schedule in dense traffic, driving safely according to traffic regulations and conditions, and providing service to passengers. Previous studies found that vehicle exhaust, long-term sitting, vibration, noise and other factors harm bus drivers’ physiological and psychological health [8,9,10,11]. For instance, Wang and Lin [10] performed a case-control study in Taipei, and found that hypertension rates among bus drivers (56.0%) in Taipei, China were much greater than that of skilled workers (30.6%) after adjustment for age. Mental illness and chronic medical conditions (e.g., cardiovascular and pulmonary diseases) may add the risk of the development of heat-related illness during extreme heat exposure [2,3].

The 2007 Intergovernmental Panel on Climate Change (IPCC) report states for the next several decades, heat waves are expected to be more intense and/or frequent [12]. The climate of China is changing in response to the overall warming trend [13,14]. Annual mean surface air temperature of China experienced a warming of 0.79 °C from 1905 to 2001, with a warming rate of 0.08 °C per 10 years [15]. China’s National Assessment Report on Climate Change noted “in the future for 20–100 years, the surface air temperature in China will continue to increase” [15]. Annual mean extreme hot days are increasing in the capital cities of China, and the increase is attributable to the changing climate [16,17]. Due to the accelerated process of urbanization and the urban heat island effect, the well-being of urban residents is severely affected by the exceptional hot weather [18,19].

Jinan, the capital city of Shandong Province in eastern China has more than 6 million inhabitants. Summer in Jinan lasts from May to September. Days with daily maximum temperature are higher than or equal to 35 °C usually appear in June, July and August. Since the 1990s, severe summer heat has occurred more frequently and lasted longer in Jinan [20]. According to the 2011 Statistical Yearbook of Jinan City, the summer daily maximum temperature has increased continuously in recent years and once was 41.4 °C in the summer of 2009 [21]. Affected by a Western Pacific subtropical high, the air humidity in this city during July and August is oppressively high. During 27–29 July of 2012, in three consecutive days the daily maximum temperature was higher than or equal to 36 °C, and the mean relative humidity was more than 66% [22]. A recent case-crossover study showed that in the summer of 2010, all four heat waves were associated with increased daily hospital visits for mental illness in Jinan, with OR values of 2.224 (95% CI 1.980–2.498), 2.940 (95% CI 2.444–3.536), 3.165 (95% CI 2.657–3.771), and 3.019 (95% CI 2.476–3.681), respectively [23]. Research on heat warning systems has paid a lot of attention to the threshold temperatures for triggering heat warnings and advice for general public [24,25]; however, few [26] have focused on the appropriateness of implementing that advice among environmental heat exposed occupational groups. In Jinan, bus drivers work in shifts. During a workday, most drivers usually drive for about eight hours (but this may vary with different bus route); and they can take short break after finishing a round of driving until the scheduled time for another loop.

Factors influencing human heat vulnerability such as age, sex, education and income have been widely studied [1,2,3,27,28,29]; but little is known about the occupational dimensions of human sensitivity to unfavorable hot weather, particularly among urban bus drivers. In order to understand the heat environment, potential effective adaptive measures and specific barriers for bus drivers during hot work days, the exploration of views and attitudes toward extreme heat and its health effects is important. Aimed at exploring aspects related to coping with heat, and ultimately develop targeted advice and policy interventions for city bus drivers, a qualitative design was used to examine bus drivers and their managers’ heat risk perceptions in the city of Jinan.

## 2. Methods

In this study, qualitative focus groups with professional bus drivers and semi-structured interviews with their managers were conducted in Jinan, China. This study is supported by the National Basic Research Program of China. Ethical approval was granted by Chinese Center for Disease Control and Prevention Ethical Review Committee (NO. 201214).

### 2.1. Study Population

All professional bus drivers in Jinan city are employees of the Jinan Public Transportation Company, from which our participants were selected. This company consists of six branch companies, a Workers’ Hospital, a Worker Training Center (WTC), and several managerial departments. About 1,000 bus drivers staff each subsidiary company, and more than two-thirds of them are males. Each branch company has about eight teams of bus drivers, and is usually equipped with either air-conditioned or “ordinary buses” (buses with no air-conditioners, and with only fans). Currently, the whole city has about 200 bus routes, where only about 20 percent of them are served by air-conditioned buses.

### 2.2. Sampling

Working environment (the cab) is an essential factor which influences drivers’ exposure to summer heat. Considering that the protective role of air-conditioning during heat episodes has been well-established [30], bus drivers were classified into two categories according to their working environment (air-conditioned and non-air-conditioned). Most of the individuals selected were drivers of “ordinary” buses, as the effects of heat are more severe in their work situation. Three branch companies were contacted to provide drivers of different teams and gender if possible. Branches contacted included: Tramway Branch Company (TBC), Hengsheng Branch Company (HBC), and Bus Rapid Transit Branch Company (BRT); of them one (TBC) has “ordinary” buses and two (HBC, BRT) have air-conditioned buses. TBC provided three teams of bus drivers (the 4th, 6th, 7th team), and each of HBC and BRT provided one team. The aim was to diversify the drivers, and to increase the chance of gathering different perceptions and attitudes. The bus driver inclusion criteria in this study were: (1) regular employee; (2) at least one year of bus driving experience. From the five teams the company provided, drivers who met the inclusion criteria were invited to participate in a semi-structured focus group.

As for manager interviews, persons with professional experience in dealing with bus driving emergencies or policymaking were selected from the company’s managerial departments. Those invited to participate included: bus team leaders; safety insurance department officers; operation department mangers; and technical maintenance personnel. In addition, medical practitioners from the Workers’ Hospital who have professional (health service) background were invited for personal interviews.

Information about this study was provided to all of our respondents, and anonymity and confidentiality were assured.

### 2.3. Data Collection

The focus groups and interviews were conducted during September 2012, during the late summer in Jinan. A total of 51 participants were recruited: 37 bus drivers, 12 managers and 2 medical staff (respondent categories are in Table 1). A total of four groups of bus drivers were held with between eight and ten participants in each. Three groups (Group 1–3) were held in a meeting room at the TBC, and one was held in a meeting room at the WTC (Group 4). Twelve managers joined in the group interview conducted in a meeting room at the HBC. Two medical staff (a doctor and a nurse) participated in face to face personal interviews at their work place.

Two different topic guides were used for the bus driver focus and manager group interviews. Both of the two topic guides were developed through reviewing the relevant literature, consulting experts from relevant fields, and drawing on the previous experience of the research team. For the driver groups, the topics covered concerns about the current warming trend; the adverse health effects they experienced during hot summer; perceptions of their own vulnerability to heat (e.g., whether they recognize themselves as more susceptible to hot weather than others); preventive actions taken during extremely hot days and attitudes toward their effectiveness; major impediment or obstacles to heat relief when driving; the resources urgently needed (e.g., timely weather information, protective knowledge, medical services, or psychological consultation). For the manager group interviews, the topics contained were the responsibilities of different departments in dealing with heat threats to bus drivers; policies made aimed at protecting the well-being of those front line employees; resources needed; and the role of the managers in the promotion of preventive actions.

**Table 1:** Respondent categories.

	**Respondents**		**Male**	**Female**
Bus driver focus groups		Bus cooling facility		
(1)	Drivers from the 4th team of TBC	Fan	7	2
(2)	Drivers from the 6th team of TBC	Fan	7	3
(3)	Drivers from the 7th team of TBC	Fan	9	1
(4)	Drivers from BRT and HBC teams	A/C ^1^	8	-
**Manager Interviews**				
Group interview	Bus team leader		3	-
	Safety insurance department officer		2	-
	Operation department manger		4	-
	Technical maintenance personnel		3	-
Personal interview	Medical practitioner (nurse, doctor)		-	2
	Total		43	8

Note: ^1^: air-conditioning.

All respondents provided written informed consent form before interviews proceeded. Each focus group or interview lasted 25–45 min; and within each topic portion, the respondents were free to develop the conversation and express their own thoughts. All focus groups and the group interview were tape recorded during the entire time. Brief notes were written during the discussions. Tape records were not required for the personal interviews since interview times were short and comprehensive notes were made. The data obtain were de-identified to assure confidentiality.

### 2.4. Data Analysis

Thematic Framework Analysis [31,32] was used to analyze the data, following five stages: familiarization, identifying a thematic framework, indexing (coding), charting, and interpretation. Firstly, the original tape-recorded voices were transcribed into text transcripts and compared with brief notes taken by recorders to ensure no information was missed. Then line by line read and re-read the textual data, carefully identifying a thematic framework, producing a list of codes representing themes identified in the transcripts. After reading the initial transcripts, the coded information closest to the research theme was identified. Ambiguous information difficult to fit into any theme was discussed by the research team to determine whether a new theme should be created. Then, using form charts with headings from the thematic framework, themes were examined searching for connections among them [33]. To ensure the themes were appropriate and all relevant views were included, the original recordings were replayed and the topic guides were applied.

## 3. Results

### 3.1. Bus Driver Focus Group Discussions

Five themes or categories were found using the bus driver group data after the analysis process to interpret the data: (1) concerns about summer heat; (2) health effects related to extreme heat; (3) adaptive measures; (4) barriers in implementing coping actions; and (5) suggested interventions. Examples of quotations (Q1, Q2…) are shown in Table 2.

**Table 2:** Illustrative quotes for each theme.

No.	Quote	Sex	Cooling facility
Theme 1, Concerns about summer heat			
Q1	“*Summer time is unbearable…the situation is getting worse than in old days…*”	F ^1^	Fan
Q2	“*The weather won’t cool down till October…definitely prolonged…*”	M ^2^	A/C
Q3	“*I saw heat warnings on TV every day…*”	M	Fan
Q4	“*You sweat heavily just sitting and resting, not to mention driving a bus.*”	F	Fan
Q5	“*We’ve got a thermometer on each bus…when outside temperature reaches 40 °C, the temperature inside the bus can be over 50 °C…*”	M	Fan
Q6	“*I’m totally not worried about the changing climate… I don’t think I can do anything about that…* ”	M	Fan
Q7	“*Warmer weather may not pose much threat to me, but I’m afraid older people will suffer more…*”	M	A/C
Q8	“*I prefer summer to winter, I never felt too hot…*”	F	Fan
Theme 2, Health effects related to extreme heat			
Q9	“*We are definitely at higher risk than other people…*”	M	A/C
Q10	“*You can stay at a cool place when it’s hot, but we can’t…*”	M	Fan
Q11	“*A driver passed out on the bus on an extremely hot day, and we had to make emergency calls to send him to hospital immediately.*”	M	Fan
Q12	“*It’s unlikely not getting sick for two consecutive summers…*”	M	Fan
Q13	“*I’ve been hospitalized for kidney stone operation twice this year, and I think the major reason is insufficient water intake…*”	M	Fan
Q14	“*My face turned hot and red because of the direct sunlight…*”	M	A/C
Q15	“*I sweat through the moment I got on the bus…keep wiping and wiping… my face hurts a lot…*”	M	Fan
Q16	“*I was very irritable when driving on very hot days…*”	M	A/C
Q17	“*I received more hostility from our passengers during hot days, and I felt hard to control my temper…*”	M	Fan
Theme 3, Adaptive measures			
Q18	“*Air-conditioning really works in summer….*”	M	A/C
Q19	“*The company provided us kinds of fluids such as tea, mineral water and soup…*”	F	Fan
Q20	“*I turned on the fan the moment I sat on the bus, and turned it off till my work shift was over…*”	M	Fan
Q21	“*I do bring some medication with me when it’s too hot…*”	M	Fan
Q22	“*We’ve got TV at the restroom at the bus terminals, weather information are easy to get…*”	M	Fan
Q23	“*We make jokes to each other and I feel better when I heard the laughter…*”	M	A/C
Q24	“*It’s nice to see our managers …delivering us a bottle of water…*”	M	A/C
Q25	“*No matter what they do…It’s still hot…*”	F	Fan
Theme4, Barriers in implementing coping actions			
Q26	“*Windows cannot be fully opened…ventilation is too poor…*”	M	Fan
Q27	“*We can’t drink as much as we want to…I’ve seen a female driver stuck in a traffic jam, with a full bladder, cried helplessly…*”	M	Fan
Q28	“*I’m afraid nothing worse than stuck in a traffic jam…*”	M	A/C
Q29	“*At rush hour, I may not have time to get off the bus and have a rest, for another round is demanded.*”	F	Fan
Q30	“*Nothing makes things any better…I already get used to this working condition…*”	M	Fan
Q31	“*I don’t think a fan would make any difference, the cab was filled with hot airflow…sometimes, a fan just makes things worse…*”	F	Fan
Q32	“*Facing the air conditioning for several hours every day is not a pleasant experience…*”	M	A/C
Q33	“*Many of us, even some young bus drivers, were diagnosed with coronary heart disease, hypertension…*”	M	Fan
Q34	“*I usually won’t ask for a sick leave…I keep working even when I feel uncomfortable, for salary’s sake…*”	M	Fan
Theme 5, Suggested interventions			
Q35	“*We all want air-conditioned buses…and that passengers also prefer A/C buses even they paid more money for it*”	F	Fan
Q36	“*Our schedule is the same no matter it’s in winter or it’s in summer…I think lesser work hours in summer will help a lot…*”	M	A/C
Q37	“*Older drivers cannot withstand the young workers’ workload…*”	M	A/C
Q38	“*I don’t think I can work till 60…I want to get retired earlier…*”	M	Fan
Q39	“*The situation cannot be improved, so raising our salary might be more encouraging…*”	M	Fan
Q40	“*We used to drive on a shaded road, it’s cool and the sun would not pour through those thick leaves…*”	M	Fan

Notes: 1 = Female; 2 = Male.

#### 3.1.1. Theme 1, Concerns about Summer Heat

The majority of the respondents believed Jinan was warmer in recent years than in previous years, and the number of hot days is increasing (Q1–2). They also recalled frequent heat warnings from the city meteorological department during this summer (Q3). As mentioned by most of the bus drivers in groups 1, 2 and 3, the temperature inside the bus is much higher than outside (Q4–5).

Most respondents barely showed any concern about the changing weather when the host came up with this question: “Do you feel worried about the current warming trend?” They saw the regional summer heat as a threat, but the “changing climate” globally is not much of their concern (Q6). Few showed care concerning the elderly; they stated that the aged are more likely to be affected by heat (Q7). Very few expressed fondness for hot weather (Q8).

#### 3.1.2. Theme 2, Health Effects Related to Extreme Heat

The majority of the respondents acknowledged that bus drivers, especially those who drive buses with no air-conditioners, were adversely affected by the heat; and they saw themselves as more susceptible than the average population largely due to the unavoidable prolonged heat exposure (Q9–10).

“Heat-related illness” was mentioned by most respondents to be a very common experience during hot days, and some noted “other drivers passed away because of heart attack” when they were driving (Q11–12). One mentioned “renal disease” due to inadequate fluid intake (Q13). Many respondents found that direct sunlight exposure and constant wiping due to heavy sweat hurt their facial skin (Q14–15). A large proportion of the drivers reported “diarrhea” during hot days, due to “irregular mealtimes” and “easily rotten food in high temperatures”.

Most respondents expressed emotional disturbance caused by extreme heat. Although they admitted they were “sleepy” and “fatigued” during any season, they noted worse feelings in hot summer weather. Terms such as “angry” “worried”, “anxious”, “agitated”, “depressed”, “absent-minded” were constantly used to describe their psychological response to high temperatures (Q16). They acknowledged that both passengers and drivers were testy when it’s hot, so the conflicts between riders and drivers happen more frequently (Q17).

#### 3.1.3. Theme 3, Adaptive Measures

Most drivers reported behavior changes during heat episodes, including “using fan or A/C” when driving, “more fluid intake”, and “using sun visor” or “sunglasses” to limit sun exposure (Q18–20). The clothes they wear are designed and required by the company. No complaints about the clothes were heard through the four discussion groups. It was said the Workers’ Hospital distributed medication named Huoxiang Zhengqi Liquid (made from Chinese herbs with the effect of lighten the symptoms of heat-related conditions) to drivers when it’s too hot; and some claimed that they would use it (Q21). Most of the drivers stated they watch weather forecasts during hot days: “TV”, “radio broadcast”, and “mobile phone messages” were the usual ways they received weather information (Q22). Some respondents stated that the nice relationship among colleagues helped cool their emotions (Q23); however, when talking about the role of “manager inspiration”, different opinions were given (Q24–25).

#### 3.1.4. Theme 4, Barriers in Implementing Coping Actions

The drivers identified several obstacles, including: poor ventilation on the bus, especially when the bus is “crowded by passengers” (Q26); lack of accessibility to the lavatory causing fluid avoidance (Q27); traffic congestion on bus routes that prolonged their working hours (Q28); inadequate rest time during the work day, and little during rush hour (Q29). A majority of the drivers had a negative attitude toward the implementation of behavioral preventive actions, and terms such as “useless” and “nothing would help” were used (Q30). The fan on the bus, despite being used “every summer day”, was said by many respondents to worsen the heat (Q31). Drivers driving air-conditioned buses also showed few unsatisfactory opinions about their work environment because of the long-time constant cool airflow (Q32).

Another issue discussed is that some young drivers were diagnosed with chronic diseases, such as “coronary heart disease” and “hypertension”, which make them more vulnerable to heat (Q33). Many participants reported that even if they felt uncomfortable when driving, they had to continue until they finish a day’s work; otherwise, passengers would complain and they might incur deductions on their salaries for sick leave (Q34).

#### 3.1.5. Theme 5, Suggested Interventions

The majority of respondents driving ordinary buses (group 1, 2 and 3) expressed their strong desire for air-conditioned buses. They point out that this may be “the best way” to improve the quality of their work environment, and that “passengers prefer air-conditioning buses as well” (Q35). Some drivers suggested the importance of a change in work schedules during the summer, which means reducing the frequency of the buses during hot weather, so their rest time will be increased (Q36). Many drivers said the aged drivers deserve better treatment as their health conditions are poorer (Q37). They asked for an earlier retirement, as they have “reached their limits” when they are in their 50 s (Q38). Other suggestions, including “salary raise” and “Road Greening” (more roadside trees) were widely acknowledged by our respondents (Q39–40).

### 3.2. Manager Interviews

Quotes for manager interviews are shown in Table 3. Most managers stated they pay a lot of attention to the adverse health effects heat posed to the bus drivers (Q41). Many of them were concerned about physiological problems during hot episodes (Q42). Some managers emphasized psychological issues brought about by heat (Q43). The majority of the managers would like to do more for the bus drivers, but this was prevented by a “tight budget” (Q44). The idea that buses without air-conditioners should be replaced by air-conditioned buses was generally appreciated (Q45); however, air-conditioned buses are “more expensive” and “energy-consuming”. It was proposed that “bus drivers” should be added to the list of “special-type-of-work”, receiving special benefits (Q46).

Medical practitioners from the Workers’ Hospital stated that a physical examination was provided once every two years. As noted by the doctor, “many of them” were diagnosed with “hypertension”, “heart problems” or other chronic diseases, and most are middle aged adults (Q47). It was suggested professional guidelines for summer heat are required, since the tips they distributed to bus drivers were mainly downloaded from the internet (Q48–49).

## 4. Discussion

### 4.1. Heat Risk Awareness among Occupational Drivers

In this study, attitudes toward climate change and summer heat in Jinan were similar for drivers from different groups. Most bus drivers confirm a warming trend in Jinan was occurring; however, they were not worried about the changing climate. Based on the response to several questions, it was clear that almost all bus drivers saw themselves especially vulnerable to extreme heat. The major reason is that they are exposed for long periods to excessively high temperatures during summer work days.

**Table 3:** Illustrative quotes for manager interviews.

No.	Quotes
Q41	“*Heat is harmful…we remind drivers to have good rest and avoid alcohol the day before if there would be a heat alert…*” (Safety insurance department officer)
Q42	“*Some aged driver had a heart attack when they were driving…stop the bus immediately…send him to the hospital…*” (Operation department manager)
Q43	“*Drivers’ performance can be influenced by their mood to a large extent…they are more likely to be agitated in hot weather…and you’ll not expect a pleasant ride…*” (Bus team leader)
Q44	“*Frankly speaking, money is the major problem…air-conditioned bus is way more expensive…*” (Technical maintenance personnel)
Q45	“*We want to purchase more air-conditioned bus…It’s healthier for them…*” (Operation department manager)
Q46	“*We expect specific policies or regulations from the government to increase drivers’ welfare…*” (Bus team leader)
Q47	“*Drivers get physical examinations once every two years…according to their health records…you might think heart disease happens to old people only, but you are wrong…*” (Medical practitioner, doctor)
Q48	“*We handed out some tips to help them better coping with heat…downloaded from the internet…*” (Medical practitioner, nurse)
Q49	“*In case of emergency while driving, professional guidelines should be provided to our drivers…*” (Medical practitioner, doctor)

To avoid morbidity and mortality, it is crucial for drivers to recognize the differences between heat stroke and milder forms of heat-related illness early and take appropriate actions. Working conditions for bus drivers can be similar, but their health status and work habits may vary. Pre-existing illnesses, either acute infections such as diarrhea or chronic diseases are risk factors that make some of the drivers more vulnerable to heat [19,34,35]. Most drivers stated that, restricted by the nature of this job, they kept working even when they were feeling “headache”, “breathless” or “dizzy”; therefore, it is possible that early symptoms of potential fatal heat stroke [3] may be overlooked. In addition, more attention should be paid to psychological health effect brought about by heat. Other studies have indicated that heat waves are associated with mental health problems [23,36,37]. The quality of bus driving not only directly affects the smoothness of the public transportation but also has great impact on citizens’ life and safety. Irritability and loss of coordination are common symptoms of heat exhaustion [3], and it is extremely dangerous particularly when it happens to bus drivers.

### 4.2. Adaptation and Barriers

In coping with heat, most bus drivers may take preventive measures; however they have encountered several barriers (as shown in Table 4). Restrict by the work condition, many drivers seldom paid any attention to health protective advices. Lack of accessibility to the lavatory is a huge obstacle. Most bus drivers do bring a bottle of water with them; however, the road situation can be very complicated and they might not be able to go to the toilet when they want [38]. Although they were informed to keep hydrated when it’s hot, intentional inadequate fluid intake was very common among bus drivers. During rush hours, the average speed of buses is only 16.5 km per hour in Jinan city [39]. Another obstacle makes working on a bus especially challenging is short rest time at rush hours, since caught in a traffic jam will definitely increases heat exposure for drivers.

**Table 4:** Preventive measures and barriers in implementation.

Preventive Measures	Barriers
Preventive tips/health knowledge	Often paid no attention or negative attitude towards their effectiveness
Water provided by bus company	Lack of accessibility to lavatory when driving/Intentional fluid intake avoidance
Fan on the bus	hot and humid work environment
Restroom with air-conditioning	short rest time at rush hour/tight schedule

The beneficial roles of cooling facilities (working fans and air-conditioning) during extreme heat were addressed in many other studies [30,40,41]; however, the benefits and harm of using fans on the bus in severe heat remain uncertain. Keatinge [42] referred to fans as a preventive measure for heat injury; but the US Centers for Disease Control and Prevention noted that “increased air movement (e.g., fans) has been associated with heat stress when the ambient temperature exceeds approximately 100 F (37.8 °C)” and that “fans should not be used for preventing heat-related illness in areas with high humidity” [43,44]. A majority of drivers stated that the temperature in the cab can be over “45 °C” during extremely hot days, and that the ventilation is still poor despite open windows when driving. Motors radiate heat and crowded passengers aggravate the comparatively enclosed hot environment. Thus, considering the high humidity weather in the summer of Jinan [20,21,22], it may be dangerous that most drivers still saw electronic fans as protective in certain circumstances [3].

### 4.3. Potential Effective Adaptive Strategies

In dealing with extreme heat, several suggested interventions were drawn from the study for policy makers to consider. Drivers suggested that the finest way to cope with heat may be replacing ordinary buses by air-conditioned buses. Although the discussion with the air-conditioned bus drivers implies that temperature in the cab of an air-conditioning bus may still be high, the benefits of using air conditioning [40] were generally acknowledged. The company did care about the productivity of the drivers, and that’s why managers consider more the cost of air-conditioned buses; however, they needed to rethink the cost for operating non-air-conditioned buses, considering medical fees and labor losses. A study on Jinan residents trip mode found a significant increase in public transportation: the passenger transportation share rate of bus in 2009 (24.44%) was 10 percent higher than that of 2004 (14.44%) [45]. Considering the public service function of buses, passengers’ feeling about the different bus types should be taken into consideration since they are very important stakeholders.

It was suggested that shade trees alongside the roads may provide protection for bus drivers. Urban shade trees offer great benefits in improving urban air quality and limiting the urban heat island effect [46]. Roadside greening is not just a way to beautify the cityscape; more importantly, it provides shade for people on the road. As mentioned by bus drivers, they felt very different when the road was shaded, and it was definitely cooler. Spending most of their working hours on the road, city bus drivers may benefit from Roadside Greening because of lesser sunburn.

### 4.4. The Role of Managers in Dealing with Heat

Another finding is that managers play an important role in helping implement preventive actions. First, they are the company policy makers and executors. Since longer break times are especially demanded by the bus drivers, the work schedule should be reorganized by managers in the summer to minimize heat exposure. Second, managers are the sources of information: either weather forecast or health protection knowledge. The role of Workers’ Hospital was largely ignored; however, it’s reasonable to argue that, holding the health records of the bus drivers, the hospital knows who needs more care during hot weather. The potential role of medical practitioners [47,48] needs further investigation.

### 4.5. Limitations

However, it cannot be assumed that the findings of this study are applicable to all city bus drivers. First, due to the convenience sampling method, the three subsidiaries which we contacted to provide bus drivers may not have chosen people in rather poor health status. Thus, those at high risk in hot days may be under-represented. Second, our research was conducted during the late summer of 2012 where it was comparatively cool in Jinan. Therefore, responses might rely on the memories of our respondents’ actions in the previous one or two months. It is possible that respondents provided some points that they thought were in the favor of the researchers. In addition, based on subjective interpretation of the initial data, the themes may vary between different researchers.

Nevertheless, this study added new important aspects of heat susceptibility that other studies may not have captured, and it provides guidance to a further quantitative survey which aims to obtain more specific findings. In China, summer heat in cities like Chongqing, Nanchang in Jiangxi Province, and Wuhan in Hubei Province could be even more severe. Jinan may not be the hottest area but it is now experiencing a significant warming [16,20,23] and residents may not well prepared for the coming frequent extreme heat. Although summer heat in different cities may vary, the working environment (the cab) for urban bus drivers is similar. The results of our study thus add an important dimension of the growing body of work that documents the heat impacts on human health, as there are very limited reports previously about occupational health in warming temperatures.

## 5. Conclusions

This study addressed several lesser known issues in coping with extreme heat for city bus drivers. We found that summer heat poses a great threat to city bus drivers’ physiological and psychological health and knowledge about heat-related illness may protect them from dangerous heat. We confirmed air-conditioning is a strong protector in minimizing negative health effects during heat exposure.

Barriers, such as lack of accessibility to lavatories and unavoidable excessive sunlight, not only impede the implementation of behavioral adaptive measures but also enhance the negative attitudes of bus drivers towards their effectiveness. Additionally, we identified the responsibilities of managers in helping promote preventive action and disseminating protective advice. We suggest that initiatives are needed now to minimize the downside temperature effects among bus drivers in hot seasons. It is of critical importance for managers to reset the work schedule, and check air conditioners and fans when summer arrives. Health records of bus drivers may help medical professionals target those at greater risk, and keep them under observation during heat attacks. Moreover, roadside greening could be an effective protective strategy to prevent heat injuries for bus drivers. Future in-depth analysis should also be needed to better plan public health interventions and better qualify health effects of heat exposure among this special occupational group.
